# Detection of lung, breast, colorectal, and prostate cancers from exhaled breath using a single array of nanosensors

**DOI:** 10.1038/sj.bjc.6605810

**Published:** 2010-07-20

**Authors:** G Peng, M Hakim, Y Y Broza, S Billan, R Abdah-Bortnyak, A Kuten, U Tisch, H Haick

**Affiliations:** 1 Department of Chemical Engineering, Technion – Israel Institute of Technology, Haifa 32000, Israel; 2 Russell Belrrie Nanotechnology Institute, Technion – Israel Institute of Technology, Haifa 32000, Israel; 3 Oncology Division, Rambam Health Care Campus, Haifa 31096, Israel; 4 Bruce Rappaport Faculty of Medicine, Technion – Israel Institute of Technology, Haifa 31096, Israel

**Keywords:** breath, volatile biomarker, nanosensor, GC-MS

## Abstract

**Background:**

Tumour growth is accompanied by gene and/or protein changes that may lead to peroxidation of the cell membrane species and, hence, to the emission of volatile organic compounds (VOCs). In this study, we investigated the ability of a nanosensor array to discriminate between breath VOCs that characterise healthy states and the most widespread cancer states in the developed world: lung, breast, colorectal, and prostate cancers.

**Methods:**

Exhaled alveolar breath was collected from 177 volunteers aged 20–75 years (patients with lung, colon, breast, and prostate cancers and healthy controls). Breath from cancerous subjects was collected before any treatment. The healthy population was healthy according to subjective patient's data. The breath of volunteers was examined by a tailor-made array of cross-reactive nanosensors based on organically functionalised gold nanoparticles and gas chromatography linked to the mass spectrometry technique (GC-MS).

**Results:**

The results showed that the nanosensor array could differentiate between ‘healthy’ and ‘cancerous’ breath, and, furthermore, between the breath of patients having different cancer types. Moreover, the nanosensor array could distinguish between the breath patterns of different cancers in the same statistical analysis, irrespective of age, gender, lifestyle, and other confounding factors. The GC-MS results showed that each cancer could have a unique pattern of VOCs, when compared with healthy states, but not when compared with other cancer types.

**Conclusions:**

The reported results could lead to the development of an inexpensive, easy-to-use, portable, non-invasive tool that overcomes many of the deficiencies associated with the currently available diagnostic methods for cancer.

Cancer kills more than seven million people every year. The most widespread cancers in the developed world, accounting for half of the cancer deaths, are prostate, lung, and colorectal cancers for men, and breast, colorectal, and lung cancers for women (Culter, 2008; Jemal *et al*, 2008). The prognosis of a cancer patient improves considerably if the disease is discovered at an early stage, when the tumour is still localised (Hirsch *et al*, 2001; Kalogerakos *et al*, 2008). However, early detection is possible only through widespread and screening, because of the asymptomatic onset of the disease. The currently available techniques do not always fulfill the requirements for reliable discrimination between cancer patients and healthy subjects. For instance, the impact of computed tomography screening on lung cancer mortality and the benefit of the prostate-specific antigen blood test for prostate cancer screening are uncertain (Culter, 2008). Screening methods such as colonoscopy for colorectal cancer and mammography for breast cancer are very reliable, but these methods are far from ideal. Colonoscopy is invasive and extremely unpleasant to the patient (Culter, 2008; Jemal *et al*, 2008). Mammography uses X-rays which may cause radiation-induced mutations. In addition, image quality depends on the breast structure, so that it is not applicable to certain segments of the screening population, such as young at-risk women (young women with a family history of breast cancer and/or *BRCA* mutations) (Bermejo-Perez *et al*, 2008) and women with dense breast tissue (∼25% of women over the age of 40 years) (Mandana, 2003; Andrew and Srinivasan, 2008).

The analysis of volatile organic compounds (VOCs) that are linked to cancer is a new frontier in medical diagnostics because it is non-invasive and potentially inexpensive (Amann *et al*, 2007; Mazzone, 2008). The principle behind this approach is based on cell biology. In particular tumour growth is accompanied by gene and/or protein changes that may lead to peroxidation of the cell membrane species and, hence, to the emission of VOCs (Singer and Nicolson, 1972; Kneepkens *et al*, 1994; Alberts *et al*, 2002). These VOCs can be detected either directly from the headspace of cancer cells (Bajaj *et al*, 2009; Barash *et al*, 2009; Filipiak *et al*, 2010) or through exhaled breath (see Amann *et al*, 2007; Ligor *et al*, 2008; Mazzone, 2008; Bajtarevic *et al*, 2009; Horvath *et al*, 2009; Ligor *et al*, 2009 and bibliography therein), as cancer-related changes in the blood chemistry lead to measurable changes in the breath by exchange through the lung (Horvath *et al*, 2009). In this study, we report that a tailor-made array of cross-reactive sensors based on organically functionalised gold nanoparticles (GNPs) discriminates between breath VOCs of healthy controls and of patients suffering from lung, breast, colorectal, and prostate cancers. Moreover, we report on the ability of these GNP sensors to distinguish between the breath patterns of different cancer types in the same pattern analysis, irrespective of age, gender, lifestyle, and other confounding factors. We find that these results compare favourably with breath testing via a chemical analysis of the constituent compounds by gas chromatography linked with mass spectroscopy (GC-MS). The results reported in this study could lead to the development of a cost-effective, easy-to-use, portable, non-invasive tool that overcomes many of the deficiencies associated with the currently available techniques used for the diagnosis of cancer.

## Materials and methods

### Test population

Breath samples were collected after signed consent from 96 volunteers aged 30–75 years, who had not ingested coffee or alcohol for at least 12 h. These were 30 primary lung cancer patients, 26 primary colon cancer patients, 22 primary breast cancer patients, and 18 primary prostate cancer patients and 22 healthy controls. All cancer patients were tested directly after being diagnosed by conventional clinical means (various imaging techniques to locate the tumour and biopsy for final diagnosis) and before chemotherapy and/or other cancer treatments. No breath collection was carried out in the 4 days after biopsy. The 22 healthy controls matched the tested cancer patients in age and lifestyle. The clinical characteristics of the study population are listed in Table 1. Additional breath samples were collected from 59 healthy volunteers, aged 20–75 years, for studying the effect of various confounding factors (Table 2). All experiments were approved by and performed according to the guidelines of Technion's committee for supervision of human experiments (Haifa, Israel).

### Breath collection

Exhaled breath was collected in a controlled manner from individuals with cancer in the same room/atmosphere and from healthy subjects. The inhaled air was cleared of ambient contaminants by repeatedly inhaling to total lung capacity for 3–5 min through a mouthpiece (purchased from Eco Medics, Duerten, Switzerland) that contains a filter cartridge on the inspiratory port, thus removing >99.99% of exogenous VOCs from the air during inspiration. Immediately after the lung washout, subjects exhaled through a separate exhalation port of the mouthpiece against 10–15 cm H_2_O pressure to ensure closure of the vellum so that nasal entrainment of gas is excluded. Complementary experiments optimising the breath collection procedure have shown that the sampling methodology simply measures alveolar breath uncontaminated by upper airways release and exogenous compounds. Exhaled breath is a mixture of alveolar air and respiratory dead space air. The dead space was automatically filled into a separate bag and the alveolar breath into a 750 ml Mylar sampling bag (purchased from Eco Medics). It should be emphasised that the described breath collection is a single-step process that does not require the volunteer to take care of changing between the dead space and alveolar breath bags. The Mylar bags used in this study are made from polyvinyl fluoride, which is chemically inert with respect to most compounds in the breath. The Mylar bags were re-used and thoroughly cleaned before each use with flowing N_2_ (99.999% purity) gas for 5–8 min (Notably, GC-MS/solid-phase microextraction (SPME) has shown that this purification process eliminates >98% of contaminants and/or VOCs from the previous sample tested in a specific Mylar bag.). At least two bags were collected per test person for analysis with GC-MS and/or for analysis with a nanosensor array based on organically functionalised GNPs – see below. All bags were analysed within 4 days from the time of breath collection, much before the allowed 3-weeks storage period, after which, according to our control experiments, the samples might start to deteriorate.

### Design and fabrication of the nanosensor array

**Figure 1:**
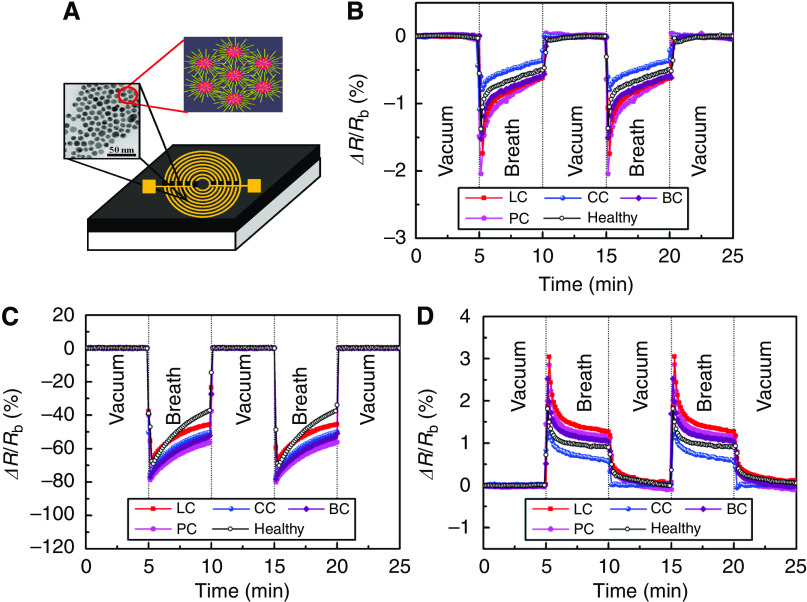
(**A**) Schematic representation of the GNP sensors in this study (not drawn to scale). The sensors were formed by successively drop casting the solutions of the molecularly modified GNP solutions onto 10 pairs of pre-prepared Ti/Au-inter-digitated electrodes. The left inset in the sensor's schematics shows a tunnelling electron micrograph (TEM) of the NPs, which connects the electrodes and forms multiple paths between them. The right inset of the sensor's schematics shows schematics of films based on molecularly modified GNPs. In these films, the metallic particles provide the electric conductivity, and the organic film component provides sites for the sorption of analyte (guest) molecules. In addition to their role as an adsorptive phase, the presence of well-defined organic spacers (i.e., capping molecules) allows a control over the inter-particle distance, and thereby, obtaining nearly uniform inter-particle distances in the composite films. This allows achieving controlled signal and noise levels. Typical resistance responses, Δ*R*/*R*_b_, of three GNP sensors functionalised with (**B**) 2-ethylhexanethiol, (**C**) decanethiol, and (**D**) 2-mercaptobenzoxazole, upon exposure to breath of a lung cancer (LC), colon cancer (CC), breast cancer (BC), and a prostate cancer (PC) patients, as well as a healthy subjects, are shown as representative examples. *R*_b_ is the baseline resistance of the GNP sensor in vacuum, and Δ*R* is the baseline-corrected steady-state resistance change upon exposure of the GNP sensor to the breath sample.

Monolayer-capped 5 nm GNPs were synthesised by a modified two-phase method (Brust *et al*, 1995), combined, if necessary, with a ligand-exchange procedure. Dodecanethiol, 4-methoxy-toluenethiol, hexanethiol, 11-mercapto-1-undecanol, decanethiol, octadecanethiol, tert-dodecanethiol, 1-butanethiol, 2-ethyl-hexanethiol, 3-methyl-1-butanethiol, 2-mercaptobenzoxazole, 11-mercapto-1-undecanol, 2-mercapto-benzyl alcohol, and 3-methyl-1-butanethiol (all purchased from Sigma-Aldrich, Rehovot, Israel) were used as organic capping layers. The GNP synthesis was described in detail in the study by Peng *et al* (2009). In all, 10 pairs of circular inter-digitated gold electrodes were deposited by an electron-beam evaporator TFDS-870 (VST Ltd, Petah Tiqwa, Israel) on device quality silicon wafers capped with 300 nm thermal oxide (purchased from Silicon Quest International Inc., Santa Clara, CA, USA). The outer diameter of the circular electrode area was 3000 *μ*m; the gaps between two adjacent electrodes and the width of each electrode were both 20 *μ*m. The functionalised GNPs were dispersed in toluene by sonication and drop cast onto the electrodes. While still coated with solution, the substrate was blown dry with N_2_. This process was repeated several times to yield the desired resistance of ∼1 MΩ. The device was dried for 2 h at room temperature in an ambient atmosphere and then baked overnight at 50°C in a vacuum oven. Figure 1A shows a schematic representation of the device. Overall, 14 GNP sensors with different organic functionalities were mounted onto a custom PTFE circuit board to form the nanosensor array.

### Breath testing using the GNP sensor array

The developed nanosensor array was placed inside a home-made stainless steel test chamber with a volume of <100 cm^3^. The sampling system first evacuated the chamber, then delivered a pulse of breath from the sample bags to the GNP sensors, and then evacuated the chamber again. In a typical experiment, signals of the GNP sensor array were collected for 5 min in vacuum, then for 5 min during breath exposure, and then again for 5 min in vacuum. The cycles were repeated 3–5 times to test reproducibility. Each sensor of the array underwent a reversible change in electrical resistance when exposed to the sample breath. The responses of the different sensors were unique because of the chemical diversity of the sensor materials. An Agilent Multifunction switch 34980 (Agilent Technologies Ltd, CA, USA) controlled by USB was used to select the active GNP sensor and measure the corresponding resistance at a given time. The electrical resistance of the GNP sensors was measured using a device analyser using integration times that span at least two power line cycles. The entire system was computer controlled.

Signals collected from all sensors in the array were analysed using standard principal component analysis (PCA) and cluster analysis (Peng *et al*, 2008, 2009; Roeck *et al*, 2008). Principal component analysis is an effective method to reduce multidimensional data space to its main components and, therefore improves the human perception ability of the data. It determines the linear combinations of sensor values so that the maximum variance between all data points can be obtained in mutually orthogonal dimensions. This results in the largest variance between sensor values from the first principal component and produces decreasing magnitudes of variance from the second to the third principle components and so on.

### Breath testing by GC-MS

The collected breath was chemically analysed, that is, its constituent compounds were identified, using GC-MS (GC-6890N; MS-5975; Agilent Technologies Ltd), combined with SPME. The SPME was used for pre-concentrating VOCs in breath samples. A manual SPME holder with an extraction fibre was inserted into the Mylar bag for 20–30 min before being delivered to the GC-MS. We used a commercially available fibre with polydimethylsiloxane-divinylbenzene coating obtained from Sigma-Aldrich. The extracted fibre in the manual SPME holder was inserted into the injector of the GC (splitless mode). We used the following oven profile: 60°C, 2 min, 8°C per min to 100°C, 15°C per min to 120°C, 8°C per min to 180°C, 15°C per min to 200°C, 8°C per min to 225°C. A capillary column H5-5MS 5% phenyl methyl siloxane (30 m length, 0.25 mm i.d., 0.25 *μ*m thickness, from Agilent Technologies Ltd) was used. The column pressure was set at 8.22 p.s.i., and the initial flow rate was 1.0 ml per min. The molecular structures of VOCs were determined by a spectral library match (and not by retention time using calibration mixtures) using the standard modular set. The VOCs common for >80% of the healthy and cancer samples, as well as their abundance with experimental error, were identified using the Automated Mass Spectral Deconvolution and Identification System (AMDIS) software. Out of the common VOCs, those that showed no overlap in abundance (taking into account their experimental errors) between the studied groups were determined. The PCA algorithm was applied to this choice of VOCs.

## Results and Discussion

This study was conducted in four phases. In the first phase, exhaled alveolar breath was collected from 177 volunteers aged 20–75 years (lung, colon, breast, and prostate cancer patients and healthy controls; Tables 1 and 2), using an ‘offline’ method described by Peng *et al* (2009). This method effectively separates endogenous VOCs in the breath from exogenous VOCs, and excludes nasal entrainment. Endogenous VOCs are generated by cellular biochemical processes in the body and, thus, may provide insight into the body's function (Kneepkens *et al*, 1994; Baubach *et al*, 2005), whereas exogenous VOCs are adsorbed from the environment (Baubach *et al*, 2005). All cancer patients were tested immediately after being diagnosed by conventional clinical means, and before any treatment.

In the second phase, we designed an array of 14 cross-reactive nanosensors, based on 5-nm GNPs (Haick, 2007; Barash *et al*, 2009; Peng *et al*, 2009) with different organic functionalities (see the ‘Materials and Methods’ section), and tested its feasibility for simultaneous detection of primary lung, colon, breast, and prostate cancers. Figure 1A shows a schematic representation of the device. Arrays of broadly cross-reactive GNP sensors are ideally suited to trace the cancer odour prints directly, without identifying the constituent VOCs (Roeck *et al*, 2008). The GNP sensors combine the robustness and processing ease of inorganic materials with the sensitivity and chemical selectivity of certain organic molecules to cancer biomarker VOCs (Haick, 2007; Jain *et al*, 2007). Some of the GNP sensors have detection limits of 1–5 p.p.b. to typical cancer biomarkers (Peng *et al*, 2009), whereas the others have a detection limit down to ∼10 p.p.t. The apparent detection limit and sensitivity of these sensors were demonstrated for different compounds elsewhere (Barash *et al*, 2009; Peng *et al*, 2009). Every sensor of the array undergoes a rapid and fully reversible change in electrical resistance when exposed to the breath sample. Some of the GNP sensors show a positive resistance change, others show a negative resistance change (Figure 1B–D). The sensing mechanism in GNP thin films is still subject to controversy (Haick, 2007). In most cases, the responses are unique because of the chemical diversity of the sensor materials. Each sensor responds to various odourants, that is, to an individual subset of VOCs in a breath sample. This method distinguishes the odour print resulting from the constituent VOCs directly, without identifying the individual compounds. This black-box approach is essentially different from the chemical analysis of a breath sample by GC-MS or related methods (see below). The subtle differences in the chemical composition that distinguish the breath of healthy individuals, and lung, breast, colorectal, and prostate cancer patients from one another lead to measurable changes in the responses of the individual GNP chemiresistors (Figure 1B–D).

**Figure 2:**
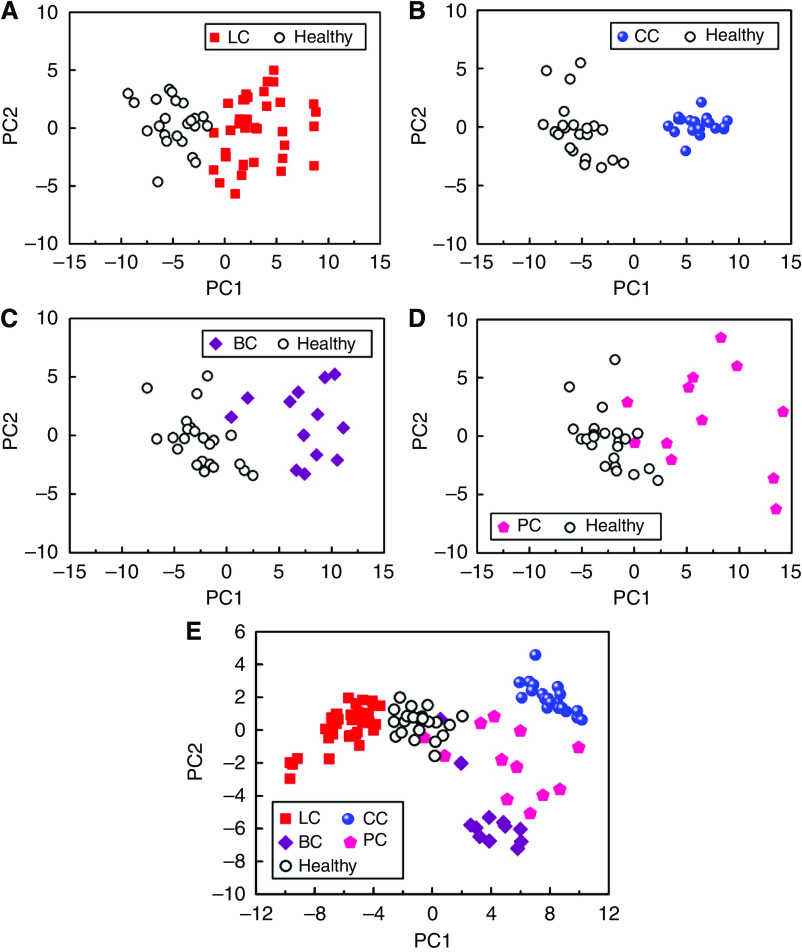
PCA plots of the GNP sensor array's resistance responses of (**A**) lung cancer (LC) and healthy controls, (**B**) colon cancer (CC) and healthy controls, (**C**) breast cancer (BC) and healthy controls, (**D**) prostate cancer and healthy controls, and (**E**) all cancer patients and healthy controls together. Each patient is represented by 1–3 points in plot. The first two principal components depicted contained >88% of the total variance in the data.

Signals collected from the 14-sensors-array after exposure to the breath of representative subjects from the 5 different test groups were analysed using standard PCA (Roeck *et al*, 2008; Peng *et al*, 2009). Principal component analysis is an effective method to improve human perception of experimental data by reducing the multidimensional data space to its main components (for more details, see the ‘Materials and Methods’ section). Figure 2 shows the maps of the first two principal components (PC1 and PC2) for each signal, which account for >88% variance. We analysed (1) lung cancer patients and healthy subjects only, (2) colon cancer patients and healthy subjects only, (3) breast cancer patients and healthy subjects only, (4) prostate cancer patients and healthy subjects only, and (5) lung, breast, colorectal, and prostate cancer patients and healthy subjects together (patient data in Table 1). A very good separation was achieved in the principal component space between the patterns of healthy controls and of patients suffering from lung, colon, and breast cancers, as seen in Figure 2A–C. Complementary analysis has not shown any correlation between the classification quality and the age of the sensors. The cluster of prostate cancer patients (Figure 2D) slightly overlaps with the healthy cluster, probably owing to the fact that many of the prostate cancer test groups have stage I cancer. Moreover, we were able to distinguish between the patterns of the four different cancers in the same principal component plot, with only minimal overlap between the prostate cancer and healthy clusters, as seen in Figure 2E. Notably, although we used the same study populations in Figure 2A–E, the clusters appear differently in principal component space, because the input data for the statistical algorithm is different. The unambiguous identification of the cancer site is a major step towards the development of a robust breath test for cancer. This study was conducted using a relatively small test population, and therefore constitutes only a proof of concept. A wider clinical study would be necessary to confirm the criteria for distinguishing different cancers, and to establish criteria for distinguishing cancer subtypes and stages (Rubin *et al*, 1997). It is reasonable to expect that clusters for a larger study population would be less well defined and some overlap would occur. In turn, cluster separation could be improved again by developing the GNP sensor array further while maintaining minimum sensitivity to potential confounding effects of common breath metabolites (such as ammonia (CAS: 7664-41-7), acetone (CAS: 67-64-1), methanol (CAS: 67-56-1), and ethanol (CAS: 64-17-5)), which, based on GC-MS/SPME, were found in approximately 20–30% of the examined patients. The iterative improvement in the sensor array while expanding the study population would eventually yield an optimised device. The use of organically functionalised GNPs allows tailoring the properties of the constituent sensors to tune their sensitivity to cancer VOCs.

In the third phase, we compared our proposed method of breath testing using a GNP sensor array with breath analysis through GC-MS. GC-MS is often used for identifying biomarker VOCs of diseases, including breast (Phillips *et al*, 2003) and lung cancers (Ligor *et al*, 2008, 2009; Mazzone, 2008; Bajtarevic *et al*, 2009; Peng *et al*, 2009), but has not been applied to distinguish between different types of cancer. In this study, we combined GC-MS with SPME. GC-MS identifies the VOCs that can serve as cancer biomarkers in breath samples and determines their relative compositions (Ouyang and Pawliszyn, 2006; Amorimb and Cardeal, 2007). SPME is a convenient method to pre-concentrate cancer biomarker VOCs in the breath samples, because GC-MS *per se* is not sensitive enough to concentrations below the p.p.m. (part-per-million) level. It must be noted that solid-phase extracts include only part of the constituent compounds, and, therefore selectively enhance the signals of certain biomarkers, whereas it potentially misses others (Barash *et al*, 2009). Hence, it is likely that not all of the cancer biomarker VOCs in the breath samples, to which the array of GNP sensors responded, were identified. For example, there is some evidence that formaldehyde is elevated in the urine (and hence blood/breath) of prostate cancer patients (Spanel *et al*, 1999) and nitrosamines in the flatulence (and perhaps in breath as is gut-produced methane) of bowel cancer patients (Amann *et al*, 2007). These may be the major compounds that are activating the sensors but they are not picked up by GC-MS/SPME.

**Figure 3:**
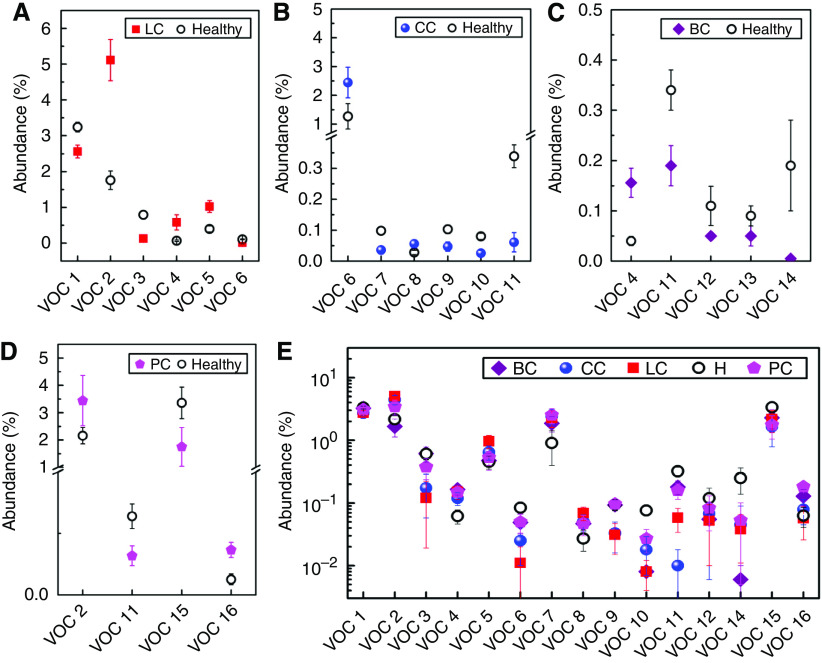
Pre-selection of the GC-MS input data for PCA: The most suitable VOCs were selected for distinguishing between healthy subjects and patients suffering from a specific cancer: (**A**) 6 of 33 common VOCs for lung cancer (LC); (**B**) 6 of 39 common VOCs for colon cancer (CC); (**C**) 5 of 54 common VOCs for breast cancer (BC); (**D**) 4 of 36 common VOCs for prostate cancer (PC). The abundance of the choice VOCs for healthy controls and cancer patients does not overlap (cf. error bars). (**E**) The entire 16 VOCs used in (panels **A–D**) for separating each type of cancer from the healthy controls. It must be noted that the GC-MS analysis did not yield compounds without overlap in abundance for lung cancer, breast cancer, colorectal cancer, and prostate cancer and healthy test groups together. The VOCs were tentatively identified as: VOC 1=1-methyl-4-(1-methylethyl)benzene (*m*/*z*=119; CAS: 99-87-6); VOC 2=toluene (*m*/*z*=91; CAS: 108-88-3); VOC 3=dodecane (*m*/*z*=57; CAS: 112-40-3); VOC 4=3,3-dimethyl pentane (*m*/*z*=43; CAS: 562-49-2); VOC 5=2,3,4-trimethyl hexane (*m*/*z*=43, CAS: 921-47-1); VOC 6=1,1′-(1-butenylidene)bis benzene (*m*/*z*=208, CAS: 1726-14-3); VOC 7=1,3-dimethyl benzene (*m*/*z*=91, CAS: 108-38-3); VOC 8=1-iodo nonane (*m*/*z*=43; CAS: 4282-42-2); VOC 9=[(1,1-dimethylethyl)thio] acetic acid (*m*/*z*=57; CAS: 24310-22-3); VOC 10=4-(4-propylcyclohexyl)-4′-cyano[1,1′-biphenyl]-4-yl ester benzoic acid (*m*/*z*=257; CAS: 82406-83-5); VOC 11=2-amino-5-isopropyl-8-methyl-1-azulenecarbonitrile (*m*/*z*=224; CAS: 93946-48-6); VOC 12=5-(2-methylpropyl)nonane (*m*/*z*=57; CAS: 62185-53-9); VOC 13=2,3,4-trimethyl decane (*m*/*z*=43; CAS: 62238-15-7); VOC 14=6-ethyl-3-octyl ester 2-trifluoromethyl benzoic acid (*m*/*z*=173; NIST: 282650); VOC 15=*p*-xylene (*m*/*z*=91; CAS: 106-42-3); and VOC 16=2,2-dimethyl decane (*m*/*z*=57; CAS: 17302-37-3). *m*/*z* indicates the major target mass.

Typically, our GC-MS/SPME analysis identifies 300–400 different VOCs per breath sample (Peng *et al*, 2009), with >87% reproducibility for a specific volunteer that was examined multiple times over a period of 6 months. Using forward stepwise discriminant analysis, we identified a number of VOCs, which are present in >80% of lung cancer patients and healthy subjects (33 VOCs) (Peng *et al*, 2009), colon cancer patients and healthy subjects (39 VOCs), breast cancer patients and healthy subjects (54 VOCs), and prostate cancer patients and healthy subjects (36 VOCs). Some of these common VOCs occur in distinctly different concentration ratios, so that the change in their overall composition can be used as a cancer biomarker (Peng *et al*, 2009). Owing to the large experimental scatter in their abundance, not all common VOCs are suitable for distinguishing between healthy controls and cancer patients. We selected the most suitable ones, for which there was no overlap in abundance between healthy controls and cancer patients – six VOCs for lung cancer, six VOCs for colon cancer, five VOCs for breast cancer, and four VOCs for prostate cancer (see Figure 3A–D and figure caption therein). To the best of our knowledge, most of the VOCs reported in this study appear for the first time in the literature. The exceptions are the two lung cancer VOCs: VOC 1 (1-methyl-4-(1-methylethyl)benzene; *m*/*z*=119; CAS: 99-87-6), which was reported earlier by Phillips *et al* (1999a, 1999b), Poli *et al* (2005), and Ligor *et al* (2009); and VOC 2 (toluene; *m*/*z*=91; CAS: 108-88-3), which was reported earlier by Poli *et al* (2005) and Ligor *et al* (2009).

From the data presented in Figure 3, we determined the characteristic odour prints of the studied cancer types through PCA and cluster analysis (see Supporting Information, Supplementary Figure S1A–D). As can be seen in the figure, good discrimination can be achieved between lung cancer patients and healthy subjects (Supporting Information, Supplementary Figure S1A) and between breast cancer patients and healthy subjects (Supporting Information, Supplementary Figure S1C). The clusters for prostate cancer patients and healthy subjects are closer together and the signature of one prostate cancer patient falls into the healthy cluster (Supporting Information, Supplementary Figure S1D). The sensitivity of this method to colon cancer is slightly lower: ∼30% of the colon cancer patient signatures are found in the healthy cluster (Supporting Information, Supplementary Figure S1B). It must be noted that the GNP array achieved the best separation for colon cancer patients and healthy subjects, illustrating the fundamental difference in the two approaches (Kushch *et al*, 2008). While achieving reasonable discrimination between healthy controls and patients suffering from one specific cancer, we were unable to determine suitable representative VOCs for distinguishing all cancers. This is because the GC-MS analysis did not yield compounds without overlap in abundance for lung, colon, breast, and prostate cancers and healthy test groups together. To achieve separation between the five study groups, we performed the PCA using all 16 VOCs used before for the successful separation of each cancer type from healthy controls, which were found in >80% of the breath samples in each study group (Figure 3E). It must be noted that one compound used to distinguish between breast cancer and healthy states (decane 2,3,4-trimethyl; CAS: 62238-15-7), was found in <80% of the breath samples in one or more of the other study groups. Hence, it was not considered in this study. Supplementary Figure S1E of the Supporting Information shows that the clusters of the five study groups are also strongly overlapping for the second set of selected VOCs. It must be noted that for any other arbitrary choice of representative VOCs, the abundances for the five study groups would overlap. Hence, it is not reasonable to expect a better discrimination through another choice of representative VOCs.

**Figure 4:**
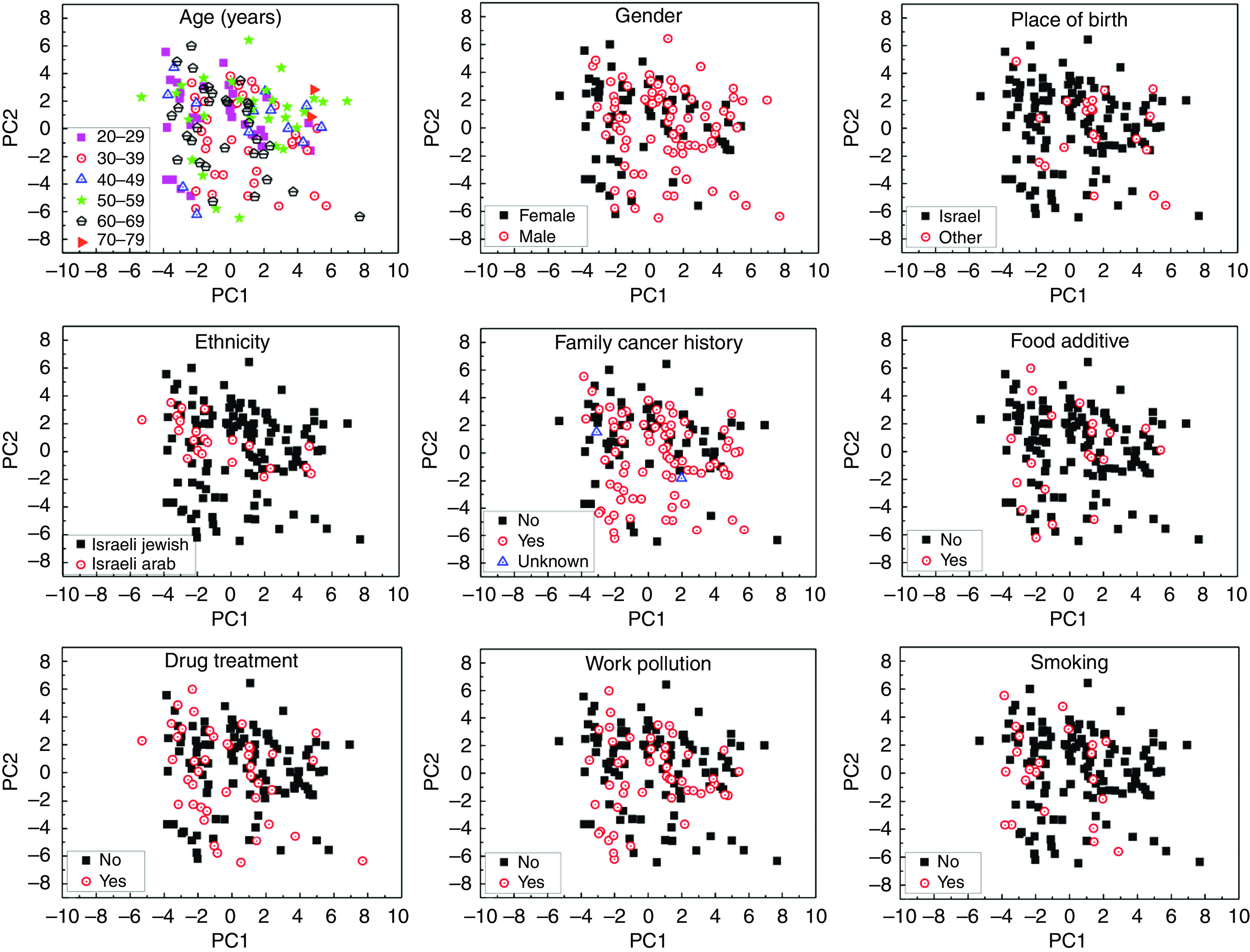
PCA plots of the GNP sensor array exposed the breath of 59 healthy subjects. Normally, two samples of each subject were analysed. Plots were analysed according to different characteristics: age, gender, place of birth, ethnicity, family cancer history, intake of food additives, drug treatment, work pollution, and smoking habits (see Table 2). The first two principal components depicted contained >90% of the total variance in the data.

Several studies using GC-MS have shown that age, gender, lifestyle, nutrition, medication, smoking habits, and other confounding factors affect the chemical composition of the breath (Mendis *et al*, 1994; Lechner *et al*, 2006; Kushch *et al*, 2008) and, as a result, the sensitivity and specificity of the diagnosis. Therefore we tested, in the fourth phase of this study, whether the same applies for GNP sensors array as well. We examined breath samples from a heterogeneous group of 59 healthy volunteers aged 20–75 years (Table 2). We found that none of the following confounding factors affected the sensor array output: gender, age, ethnic origin, family cancer history, intake of food additives, drug treatment, exposure to environmental toxins, and smoking habits (Figure 4). In contrast, we could trace and identify by GC-MS/SPME a multitude of VOCs, which can be linked to these confounding factors. For example, 2,6,11-trimethyl-dodecane (CAS: 31295-56-4) was found in 80% of the males, but in none of the females participating in the test; 3,7-dimethyl-undecane (CAS: 17301-29-0) was found in 100% of the subjects with allergies, but only in 9% of the subjects without allergies. The presence of various non-cancer-related breath VOCs in a heterogeneous study population might obscure cancer biomarker VOCs, and might be in part responsible for the observed inferior discriminative power of GC-MS/SPME compared with the GNP sensor array.

## Summary and Conclusions

Breath analysis with a GNP sensor array compared favourably with breath analysis using GC-MS because: (1) no pre-treatment of the breath samples, such as pre-concentration or de-humidification, is required; (2) the test itself is fast, easy to carry out, and does not necessarily require a trained operator; (3) no time-consuming pre-selection of the experimental data is necessary before the statistical analysis to achieve separation between the tested groups; (4) the different cancers form clusters well separated from one another and from the healthy clusters; and (5) the GNP sensor array is insensitive to various confounding factors, which strongly affect the chemical composition of the breath. The VOC signatures of the different types of cancer, with almost no overlapping among them, could lead to the development of a cost-effective, easy-to-use, portable, and non-invasive diagnostic tool for detecting lung, breast, colorectal, and prostate cancers through a single breath test, which could have a significant impact on cancer mortality through improved widespread screening. The same tool might also help to distinguish between primary cancer *vs* metastases, rapidly monitor therapy success and response to the administered treatment, as well as to detect early recurrence in an annual routine survey.

## Figures and Tables

**Table 1:** Clinical characteristics of 96 cancer patients (30 lung cancer, 26 colon cancer, 22 breast cancer, and 18 prostate cancer) and 22 healthy controls, aged 30–75 years, tested for this study;^a^ the overall ratio between male-to-female volunteers is ∼1 : 1.^b^

**Cancer type**	**GC-MS**	**Sensor array**	**No. of patients**	**Smoker (Y/N)**	**Ex-smoker (Y/N)**	**Histology**	**Cancer stage**	**Additional data**
Lung cancer^c^	x	x	2	Y		NSCLC	3A	
	x		1	Y		NSCLC	3A	Diabetes; takes glucophage
		x	1	Y		NCSLC	3A	Ischaemic heart condition; takes plavix and aspirin
	x	x	1	Y		N/A	3B	High cholesterol levels; HTIV; takes simoville and aspirin
	x		1	N	N	NSCLC	3B	Diabetes; takes various medications
	x	x	1	N	Y	NSCLC	3B	Takes optalgin and oxycontan
		x	4	N/A	N/A	N/A	3B	
		x	3	N	Y	NSCLC	3	
	x	x	1	N	N	NSCLC	4	
	x		1	N	N	NSCLC	4	
	x	x	1	N	Y	SqCLC	4	HTIV; hyperlipidaemia; takes normiten, omnic, and simoville
	x		1	N	N	NSCLC	4	Takes normiten, simoville, teraperin, and omperdex
	x		1	Y		NSCLC	4	
	x	x	1	Y		NSCLC	4	
	x		1	N	N	NSCLC	4	Heart disease; takes various medications
	x		1	Y		N/A	4	High blood pressure; takes kaptobril
		x	1	N/A	N/A	N/A	4	
		x	1	N	N	NSCLC	4	
		x	1	N	N	NSCLC	4	Heart attack; takes valium
	x		1	N	N	NSCLC	1	Takes medications; did not perform the test adequately
	x		1	Y		NSCLC	2	
		x	3	Y		NSCLC	N/A	
								
Colon cancer		x	2	Y		Tubolovillous adenoma	—	Pre-malignant
	x	x	3	N	N	Modified AC	1	
		x	2	N	Y	Rectum AC	2	
	x		1	Y		N/A	2	
	x		2	N	N	N/A	2	
	x		1	N	N	N/A	2	High blood pressure; takes various medications
	x		1	Y		Rectum AC	2	High blood pressure; takes various medications
	x		1	N	N	N/A	3	Atrial fibrilation; takes various medications
	x	x	2	N	Y	Rectum AC	3	Diabetes; high blood pressure; takes various medications
	x	x	1	N	Y	Rectum AC	3	Hyperlipidaemia; high blood pressure; takes various medications
	x	x	1	N	N	Rectum AC	3	Diabetes; high blood pressure; takes various medications
	x	x	2	N	N	Rectum AC	3	
	x		1	N	N	N/A	4	
	x		1	Y		Rectum AC	4	High blood pressure; takes normitten
	x		1	Y		Rectum AC	4	
	x	x	4	Y		NEC	4	
								
Breast cancer	x		1	N	N	N/A	1	Heart disease; high blood pressure; astrophorosis; takes various medications
	x		1	N	N	N/A	1	Thrombocytopaenia; takes various medications
		x	1	N	N	IDC	1	Gastritis; high blood pressure; takes various medications
	x		1	N	N	IDC	2	High blood pressure; hyperlipidaemia; takes cilaril plus and simoville
		x	3	N	N	N/A	3	
		x	1	N	N	N/A	3	Epilepsy; takes douplephat, lamcital, and clonax
	x		1	N	N	N/A	N/A	High blood pressure; diabetes; takes various medications
	x	x	2	N	N	IDC	N/A	
	x	x	3	N	N	IDC	2	
	x		1	N	Y	IDC	N/A	Hypoactivity of the thyroid glands; takes altroxin and vitamins
		x	3	N	N	N/A	3	
	x		1	N	N	N/A	N/A	
	x		1	Y		N/A	N/A	Several medical conditions; takes various medications
	x		1	N	N	N/A	1	Diabetes
	x		1	N/A	N/A	N/A	2	
								
Prostate cancer	x		1	N	N	AC	1	
	x		1	N	N	AC	1	Glaukoma; takes various medications
	x		1	N	N	AC	1	Diabetes; takes various medications
		x	1	N	N	N/A	1	High blood pressure; takes enaladex
		x	1	N	N	N/A	1	Diabetes; bypass; takes various medications
	x		1	N	N	AC	1	High blood pressure; takes various medications
	x	x	1	N	N	AC	1	Diabetes; high blood pressure; hyperlipidaemia; takes various medications
	x		1	N	Y	AC	1	Cardiac arrhythmia; takes various medications
	x	x	2	N	Y	AC	1	
	x	x	3	N	N	AC	1C	Several health conditions; takes various medications
	x		1	N	N	AC	2	Several health conditions; takes various medications
	x	x	1	N	N	AC	2	Diabetes; brain stroke 2-year before breath test; takes various medications
		x	2	Y		N/A	2	Back problems; takes casodex
		x	1	Y		AC	4	High blood pressure; takes enaladex and clexan
								
Healthy controls	x	x	4	N	N	N/A	N/A	
	x	x	5	N	N			
		x	2	N	N			
		x	1	Y				
	x	x	1	N	N			Subactivity of the thyroids glands; takes latroxin
	x	x	3	N	N			High blood pressure
	x		1	N	N			High blood pressure; takes blood pressure regulating medications
	x		1	N	N			Takes altroxyn
	x	x	3	Y				Diabetes
	x		1	N/A	N/A			

Abbreviations: GC-MS=gas chromatography/mass spectroscopy; NSCLC=non-small cell lung carcinoma; SqCLC=squamous cell lung carcinoma; AC=adenocarcinoma; NEC=nero-endocrin carcinoma; IDC=invasive ductal carcinoma; HTIV=human T cell immunodeficiency virus; N/A=not applicable.

a The set of GNP sensors was not influenced by characteristics, such as gender, age, or smoking habits (see Figure 4).

b Lung cancer and colon cancer patients: male and female; breast cancer patients: only female; prostate cancer patients only male.

c A GC-MS study by Phillips *et al* (2003) have not shown differences in breath signals between the stages of lung cancer. However, it is not certain that this is the case with the developed array of GNPs sensors – further experiments should be conducted before reaching a definitive conclusion in this context With this in mind, most of the lung cancer subjects in this trial were chosen to have advanced or metastatic lung cancer, even though the group chosen do not mimic the population to which the test would ideally be applied to.

No inclusion/exclusion criteria were applied in this group of volunteers.

**Table 2:** Clinical characteristics of the test population for 59 healthy volunteers, aged 20–75 years, for studying the effect of various confounding factors

	**Total number of subjects per age group (years)**						
**Age group**	**20–29**	**30–39**	**40–49**	**50–59**	**60–69**	**70–75**	**Total**
**Number**	**12**	**13**	**8**	**11**	**14**	**1**	**59**
*Gender*							
Male	3	9	2	5	9	1	29
Female	9	4	6	6	5		30
							
*Smoker*							
No^a^	9	12	8	9	11	1	50
Yes^b^	3	1		2	3		9
							
*Family cancer history*							
No	6	6	2	6	4		24
Yes	6	7	6	5	9	1	34
Unknown					1		1
							
*Drug treatment*							
No	11	13	6	5	2		37
Yes	1		2	6	12	1	22
							
*Chronic disease*							
No	10	12	7	4	4		37
Yes	2	1	1	7	10	1	22
							
*Pollutant exposure*							
No	7	10	5	7	8	1	38
Yes	5	3	3	4	6		21
							
*Allergies*							
No	9	11	3	8	10		41
Yes	3	2	5	3	4	1	18
							
*Food additive*							
No	11	13	5	10	12	1	52
Yes	1		3	1	2		7
							
*Place of birth*							
Israel	11	11	7	9	8		46
Other	1	2	1	2	6	1	13

No inclusion/exclusion criteria were applied in this group.

a The non-smoker group may include former smokers and passive smokers.

b The smoker group includes both heavy and light smokers.
