# Supplementary material for: Detection of lung, breast, colorectal, and prostate cancers from exhaled breath using a single array of nanosensors
Source: Br J Cancer. 2010 Jul 20;103(4):542–51. doi: 10.1038/sj.bjc.6605810 (PMC2939793; doi:10.1038/sj.bjc.6605810)
Supplement: Supporting information [file 6605810x1.doc]

*Supporting Information for:*

**Detection of Lung, Breast, Colorectal, and Prostate Cancers from Exhaled Breath using a Single Array of Nanosensors**

Gang Peng^1,2,§^, Meggie Hakim^1,§^, Yoav Y. Broza^1,§^, Salem Billan^3^, Roxolyana Abdah-Bortnyak^3^, Abraham Kuten,^3,4^ Ulrike Tisch^1,2^ & Hossam Haick^*1,2^

^§^ The three authors have contributed equally.

1. Department of Chemical Engineering, Technion – Israel Institute of Technology, Haifa 32000, Israel
2. Russell Barrie Nanotechnology Institute, Technion – Israel Institute of Technology, Haifa 32000, Israel
3. *Oncology Division, Rambam Health Care Campus, Haifa 31096, Israel*
4. *Bruce Rappaport Faculty of Medicine, Technion – Israel Institute of Technology, Haifa 31096, Israel*

**Fig. S1** PCA of the GC-MS/SPME analysis, using **(a)** 6 common VOCs for distinguishing LC from healthy states; **(b)** 6 common VOCs for CC from healthy states; **(c)** 5 common VOCs for distinguishing BC from healthy states; **(d)** 4 common VOCs for distinguishing PC from healthy states; and **(e)** the entire 16 VOCs used in Fig. S2(a-d) for distinguishing all five study groups. The abundances of the VOCs used are given in Fig. 3. Each point represents one test person. The first two principal components depicted contained >88% of the total variance in the data.
